# Dosimetric impact of MLC positional errors on dose distribution in IMRT

**DOI:** 10.1002/acm2.14158

**Published:** 2023-09-18

**Authors:** Hiromi Enomoto, Yukio Fujita, Saki Matsumoto, Yujiro Nakajima, Miyuki Nagai, Ayako Tonari, Takeshi Ebara

**Affiliations:** ^1^ Department of Radiology Kyorin University Hospital Mitaka Tokyo Japan; ^2^ Department of Radiological Sciences Komazawa University Setagaya Tokyo Japan; ^3^ Department of Radiation Oncology Kyorin University Mitaka Tokyo Japan; ^4^ Department of Medical Radiological Technology Faculty of Health Sciences Kyorin University Mitaka Tokyo Japan

**Keywords:** VMAT, MLC positional error, dose error, plan complexity, planning

## Abstract

Optimizing the positional accuracy of multileaf collimators (MLC) for radiotherapy is important for dose accuracy and for reducing doses delivered to normal tissues. This study investigates dose sensitivity variations and complexity metrics of MLC positional error in volumetric modulated arc therapy and determines the acceptable ranges of MLC positional accuracy in several clinical situations. Treatment plans were generated for four treatment sites (prostate cancer, lung cancer, spinal, and brain metastases) using different treatment planning systems (TPSs) and fraction sizes. Each treatment plan introduced 0.25–2.0 mm systematic or random MLC leaf bank errors. The generalized equivalent uniform dose (gEUD) sensitivity and complexity metrics (MU/Gy and plan irregularity) were calculated, and the correlation coefficients were assessed. Furthermore, the required tolerances for MLC positional accuracy control were calculated. The gEUD sensitivity showed the highest dependence of systematic positional error on the treatment site, followed by TPS and fraction size. The gEUD sensitivities were 6.7, 4.5, 2.5, and 1.7%/mm for Monaco and 8.9, 6.2, 3.4, and 2.3%/mm (spinal metastasis, lung cancer, prostate cancer, and brain metastasis, respectively) for RayStation. The gEUD sensitivity was strongly correlated with the complexity metrics (*r* = 0.88–0.93). The minimum allowable positional error for MLC was 0.63, 0.34, 1.02, and 0.28 mm (prostate, lung, brain, and spinal metastasis, respectively). The acceptable range of MLC positional accuracy depends on the treatment site, and an appropriate tolerance should be set for each treatment site with reference to the complexity metric. It is expected to enable easier and more detailed MLC positional accuracy control than before by reducing dose errors to patients at the treatment planning stage and by controlling MLC quality based on complexity metrics, such as MU/Gy.

## 1 INTRODUCTION

Volumetric modulated arc therapy (VMAT) optimizes the gantry angle, dose rate, and movement of a multileaf collimator (MLC), and uses advanced intensity modulation to deliver the required dose to the target volume while reducing that to the organs‐at‐risk (OARs) for efficient treatment. However, because of the complex modulation of the mechanical parameters, discrepancies occur between the planned and delivered movements of the mechanical parameters. Machine accuracy control is necessary to reduce discrepancies in mechanical parameters.

Previous studies have shown that MLC positional errors in mechanical parameters have the greatest impact on dose distribution. Oliver et al.^1^ reported dose errors due to the gantry angle, Monitor Unit (MU), and MLC movement errors in VMAT for prostate cancer. This study found that large variability in dose sensitivity led to MLC positional errors (>6.4% generalized equivalent uniform dose [gEUD] change). The other errors (i.e., gantry position and MU) were relatively insignificant (<0.1% gEUD change). These findings are similar to those of the other previous studies on the dosimetric impacts of MLC positional errors.^2^, ^3^ These studies suggest that the MLC positional error is one of the most important factors affecting the accuracy of dose delivery.

The dose error owing to MLC positional errors in VMAT plans depends on the MLC sequence. Oliver et al.^4^ pointed out the differences in gEUD sensitivity with MLC positional error (%/mm) between dynamic multileaf collimator intensity‐modulated radiotherapy (MLC IMRT) and VMAT. The dose sensitivity of dynamic MLC IMRT is twice that of VMAT. One possible explanation for this is that the mean MLC gap is smaller in the dynamic MLC IMRT plans. This suggests that the dose sensitivity strongly depends on the MLC sequence. Furthermore, Pogson et al.^5^ reported dose sensitivity variations owing to MLC positional errors between VMAT plans with different MLC sequences. The MLC sequence can vary depending on the clinical situation, including, for example, the treatment planning system (TPS)/MLC sequencer, treatment site, fraction size, and beam modality. However, previous studies investigated the dose sensitivity for a limited treatment site and TPS.^1^, ^2^, ^3^, ^4^, ^5^, ^6^, ^7^, ^8^, ^9^ To the best of our knowledge, no study has evaluated these factors in a combined and comprehensive manner yet. Moreover, it is not completely clear which factors strongly influence dose errors due to MLC misalignment in different clinical situations. Therefore, additional studies are required to clarify the dose errors caused by differences in the MLC sequences that depend on different combinations.

Recent studies in the field of MLC positional errors have adopted multiple approaches. Focusing on MLC parameters, the effects of MLC speed and minimum segment width on dose distribution have been reported.^10^, ^11^ Dose variation and positional accuracy due to MLC positional error for modern irradiation methods such as multiple brain metastases using linear accelerators have been studied.^12^ Other reports have also been published comparing the detection ability of MLC positional errors to tools such as the log file and EPID (Electronic Portal Imaging Devices) for detecting MLC misalignment.^13^, ^14^, ^15^ Furthermore, an algorithm has been developed recently to predict MLC position discrepancies using machine learning and its prediction accuracy has been studied.^16^, ^17^, ^18^, ^19^, ^20^ Among the mechanical parameters, dose errors due to MLC positional errors have the largest impact, and many studies have been conducted to examine its impact and prediction accuracy.^1^, ^2^, ^3^, ^4^, ^5^, ^6^, ^7^, ^8^, ^9^, ^10^, ^11^, ^12^, ^13^, ^14^, ^15^, ^16^, ^17^, ^18^, ^19^, ^20^

This study focused on the identification of the clinical situations that affect the MLC sequence and change the dose error among the various combinations of radiotherapy techniques. Recent developments in equipment and radiation oncology have increased the number of treatment sites to which VMAT and hypofractionated radiotherapy are applied. In addition, MLC sequences differ depending on the TPS used, even if the same linear accelerator is used. However, medical physicists need to know under what clinical conditions dose errors occur to apply correct doses to patients. Furthermore, although there is a broad variety of clinical situations, the positional accuracy of MLCs is often managed with reference to a single guideline value. Therefore, in addition to the verification of the effect on the dose, we considered it necessary to verify the conventional reference values from the viewpoint of MLC positional accuracy management.

The purpose of this study is to investigate the variations in dose sensitivity with MLC positional error for multiple treatment sites, TPSs, and fraction size. The next step is to quantify and elucidate the factors associated with dose sensitivity differences by comparing the complexity metrics. Furthermore, the optimum tolerance of the MLC positional accuracy is determined based on the dosimetric consequences of MLC positional errors in several clinical situations. This study will identify clinical situations vulnerable to dose fluctuations in various radiation therapy modalities. The significance of this study is to identify clinical situations vulnerable to MLC misalignment, which will allow for appropriate MLC positional accuracy control according to the radiotherapy technique being delivered.

## 2 METHODS

### 2.1 Outline

**FIGURE 1:**
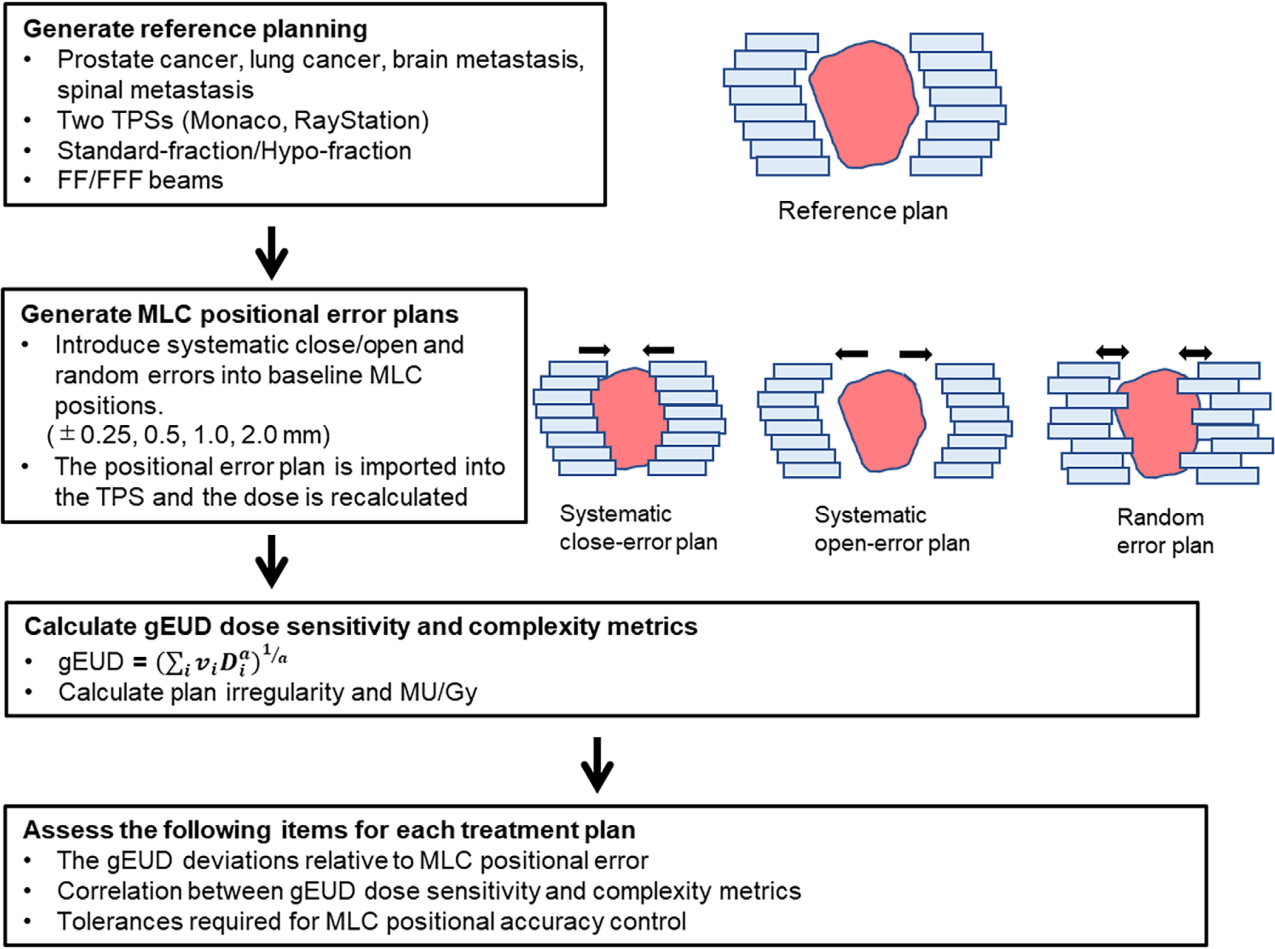
Overview of the treatment planning and multileaf collimator (MLC) positional error plan.

Figure 1 shows the outline of the study. The treatment plans were generated using different TPSs in combination with several clinical situations and used as reference plans. The positional errors of the left and right MLC systematically displaced in the closing direction (hereafter referred to as “systematic close”) and in the opening direction (hereafter referred to as “systematic open”) with respect to the MLC position of the reference plan, and the MLC random positional error plan was generated. The generated MLC positional error plans were recalculated considering the dose with TPS, and the gEUD was calculated as the index of the dose error. The correlation between gEUD sensitivity and treatment plan complexity, such as the ratio monitor units (MU)/Gy and the plan irregularity (PI), were evaluated. The gEUD sensitivity for the MLC positional error was evaluated for each treatment plan to assess the dose error with respect to the MLC positional error.

### 2.2 Clinical treatment plan

**FIGURE 2:**
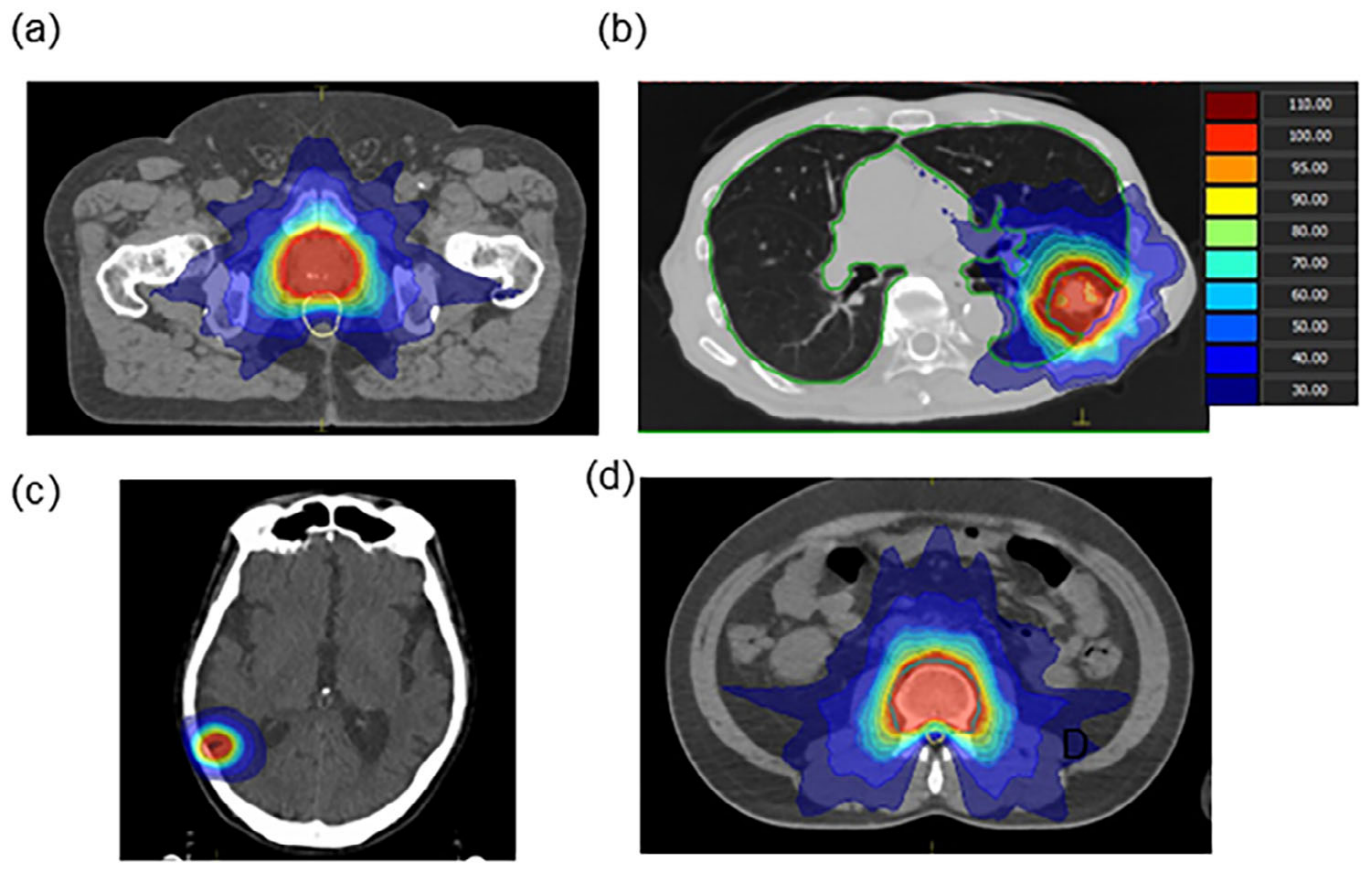
Generated clinical treatment plans. Dose distributions in reference plans for (a) prostate cancer (78 Gy/39 fractions), (b) lung cancer (52 Gy/4 fractions), (c) brain metastasis (42 Gy/10 fractions), and (d) spinal metastasis (30 Gy/10 fractions) using the Monaco treatment planning system (TPS).

In this study, 640 VMAT treatment plans were generated for prostate cancer, lung cancer, and brain and spinal metastases. The linear accelerator used in the investigation of this study was Versa HD (Elekta, Stockholm, Sweden), and energies of 6 MV and 10 MV with flattening filters and 6 MV FFF and 10 MV FFF without flattening filters were applied for treatment planning. Treatment planning and dose calculations were performed using Monaco (version 5.11, Elekta, Stockholm, Sweden) with a Monte‐Carlo‐based dose‐calculation algorithm and RayStation (version 6.3, RaySearch Laboratories, Stockholm, Sweden) with a collapsed cone‐convolution (CCC)‐based dose‐calculation algorithm. Table 1 shows the treatment sites and prescribed doses in this study. Treatment plans were generated for four treatment sites using two TPSs with two different fraction sizes and FF and FFF beams for 20 cases each, as shown in Figure 2. The treatment plans were generated based on the dose constraints shown in Table 2.

**TABLE 1:** Combination of the treatment site and prescription dose.

		Prescription dose		
Treatment site	TPS	Standard fraction	Hypofraction	Number of treatment plans
Prostate cancer	Monaco RayStation	78 Gy/39 fractions	36.25 Gy/5 fractions	80 plans 80 plans
Lung cancer	Monaco RayStation	60 Gy/30 fractions	52 Gy/4 fractions	80 plans 80 plans
Brain metastasis	Monaco RayStation	42 Gy/10 fractions	30 Gy/3 fractions	80 plans 80 plans
Spinal metastasis	Monaco RayStation	30 Gy/10 fractions	16 Gy/single fraction	80 plans 80 plans

Abbreviation: TPS, treatment planning system.

**TABLE 2:** Summary of the dose constraints of generated reference treatment plans.

Treatment sites	Target volumes/Critical structures	Dose–volume histogram (DVH) parameters	
Prostate	PTV	D_95%_, D_98%_ < 90%, D_2%_ < 110%	
	Rectum	Standard fraction	V_63 Gy_ < 17% V_21 Gy_ < 60%
		Hypofraction	V_36.25 Gy_ < 4.6% V_18.13 Gy_ < 50%
	Bladder	Standard fraction	V_63 Gy_ < 25% V_21 Gy_ < 50%
		Hypofraction	V_36.25 Gy_ < 7.8% V_18.13 Gy_ < 49%
Lung	PTV	D_95%_, D_98 %_ < 90%, D_2 %_ < 110%	
	Normal lung	V_20 Gy_ < 35%, V_5 Gy_ < 60%	
Brain metastasis	PTV	D_95%_, D_98 %_ < 90%, D_2 %_ < 110%	
	Normal brain	Standard fraction	V_38.37 Gy_ < 7 cm^3^
		Hypofraction	V_23.1 Gy_ < 7 cm^3^
Spinal metastasis	PTV	D_90 %_, D_98 %_ < 80 %, D_2 %_ < 110 %	
	Spinal cord	Standard fraction	D_max_ < 25 Gy
		Hypofraction	D_0.35 cm3_ < 10 Gy

Abbreviation: PTV, planning target volume.

### 2.3 MLC positional error plans

The MLC positional error plan was generated for the reference plan using an in‐house program in MATLAB (version 9.8, MathWorks, Natick, MA, USA). Systematic close/open positional errors of ±0.25, 0.5, 1.0, and 2.0 mm were added to the MLC position for each control point for all reference plans. Because dose errors for random MLC positional errors have been reported to be negligibly small in previous studies,^1^, ^6^, ^7^ only treatment sites with the largest systematic errors were included in the analysis. Random MLC positional errors of 0.25, 0.5, 1.0, and 2.0 mm were added to the MLC position at each control point for the spinal metastasis reference plan. The random MLC positional error was simulated by sampling a Gaussian function (0.25−2.0 mm) with a standard deviation equal to the magnitude of the error. Systematic and random positional error plans were imported into the TPS, and the dose was recalculated, generating a total of 5120 systematic positional error plans and 1280 random positional error plans.

### 2.4 Calculating the gEUD sensitivity

As the usual dose–volume histogram (DVH) assessment cannot evaluate organ‐specific biological characteristics, the gEUD^21^ (based on the biological model) was used as an index to assess the dose error. The dose and volume for each structure were obtained from the DVH of the reference plan and MLC positional error plan. The gEUD was calculated from the dose (D_i_) and volume (v_i_) using Equation (1),

(1) $$\;gEUD\;=\;\left[\sum_{i}v_{i}D_{i}^{a}\right]1/a$$

The *a*‐value in this equation is the intrinsic coefficient in tumors and normal tissues and is the index of the dose‐volume effect for a particular organ. The a‐values were determined from existing literature^7^, ^22^, ^23^ for each treatment site, that is, *a* = −25, −20, −10, and −20 for prostate cancer, lung cancer, brain metastasis, and spinal metastasis, respectively. The Burman model^24^ was used to determine the *a*‐value for the OARs. The gEUD was calculated for the target volume and OARs and the gEUD sensitivity (%/mm or Gy/mm) relative to the MLC positional error was evaluated. We also examined whether there was a significant difference in gEUD sensitivity between systematic close and open MLC positional errors for each treatment site.

Significance tests were performed for the average gEUD sensitivity among the TPSs and fraction sizes. Normality was assessed using the Shapiro–Wilk test and *p*‐values were calculated using the *t*‐test or Wilcoxon signed‐rank test. The *p*‐values were evaluated at a significance level of 0.05.

### 2.5 Assessing the correlation relationship between the gEUD sensitivity and the complexity metrics

Many metrics have been published for complexity metrics,^25^, ^26^, ^27^, ^28^, ^29^, ^30^, ^31^ and a recent review reported the relationship between complexity metrics and quality assurance outcomes.^32^, ^33^ We examined MU‐ and leaf gap‐related complexity metrics that affect dose sensitivity and the factors that affect dose error relative to MLC positional error. Previous studies have shown that dose errors due to MLC positional errors correlate with the MU and MLC gap.^1^ In this study, MU/Gy and PI were applied as complexity metrics. MU/Gy was calculated as the ratio of the total MU to the fraction size. This metric is the simplest metric that can also be checked and controlled during treatment planning. We also need to evaluate the complexity metrics for MLC aperture as the MLC sequence may differ depending on the TPS used, even if the linear accelerator used is the same. In the case of a mere MLC aperture area evaluation, the MLC aperture area may be different in the same case because it depends on the optimizer and MLC sequencer. Therefore, we applied PI, which expresses the aperture area and the noncircularity of the MLC aperture shape, reported by Du et al.,^29^ as a complexity metric. PI is the average of aperture irregularity per control point and beam irregularity weighted by segment MU. PI was calculated for the reference plan using MATLAB.

Correlations between the calculated gEUD sensitivity and complexity metrics were evaluated using linear regression analysis. Spearman or Pearson correlation coefficients and *p*‐values were calculated after assessing normality using the Shapiro–Wilk test. Correlation coefficient values ≤0.2 were considered as zero correlation, values from 0.2 to 0.4 as a weak correlation, values from 0.4 to 0.7 as a moderate correlation, and values >0.7 as a strong correlation. The *p*‐values were evaluated at a significance level of 0.05.

### 2.6 Determination of MLC positional accuracy required for accuracy control

The MLC positional error that can maintain the gEUD change rate with MLC positional error within ±2% of the target volume was calculated as the acceptable value required by the MLC positional accuracy control in the combination of the treatment site, TPS, and fraction size. This threshold was set as a value that could be measured using a standard dosimeter with a dose error larger than ±2% for clinical situations, as suggested by Blake et al.^3^

## 3 RESULTS

### 3.1 Assessment of the dose distribution for the reference plan and the MLC positional error plan

**FIGURE 3:**
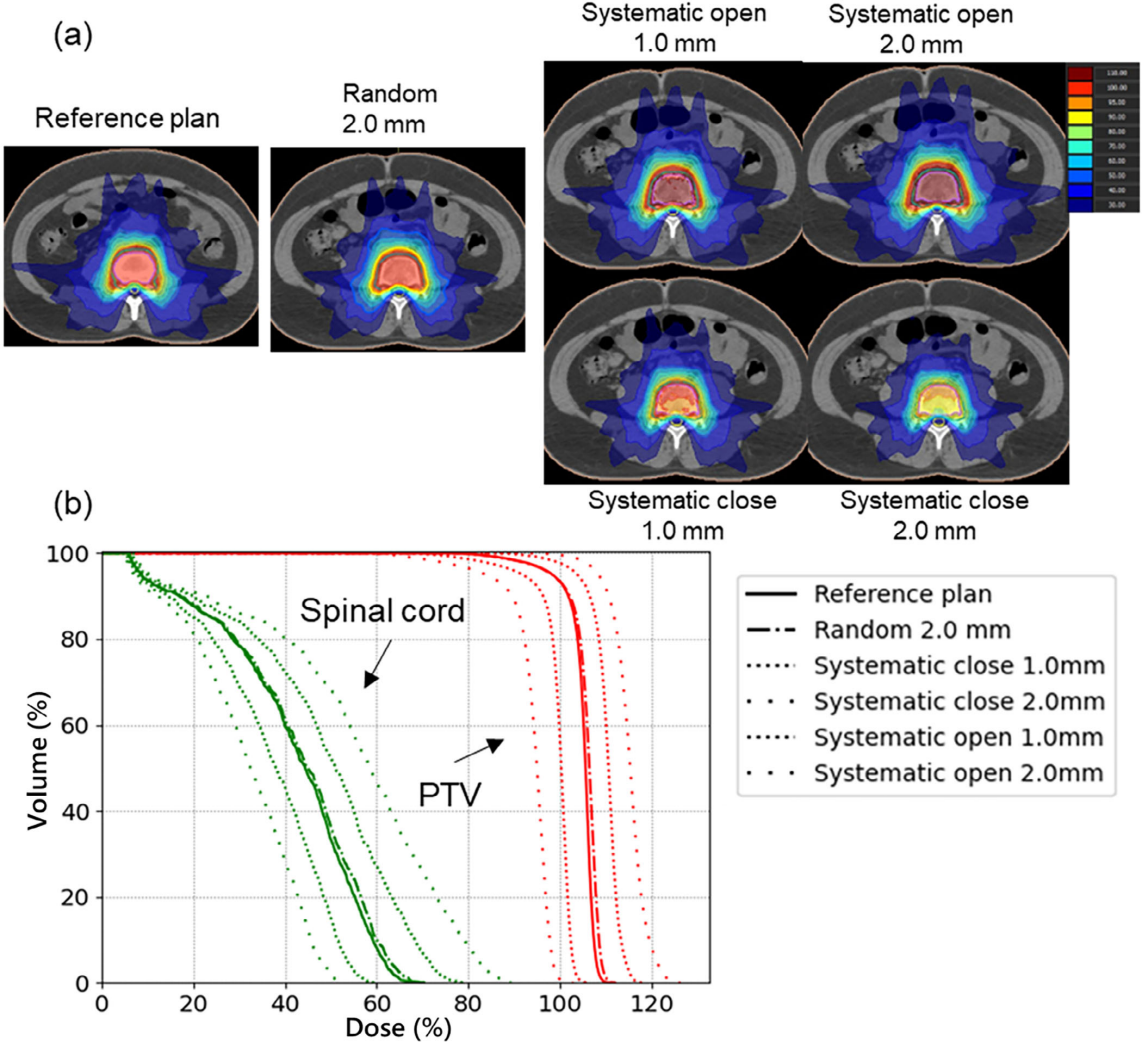
Examples of dose distributions and dose–volume histogram (DVH) for the reference plan with the standard fraction using the Monaco TPS and the plan with MLC systematic close/open and random position errors. (a) Dose distributions and (b) DVH for MLC systematic close/open positional errors (±1.0 and 2.0 mm, respectively) and random positional error of 2.0 mm for spinal metastasis. As the dose distributions for the plans with systematic positional errors of ±0.25 and 0.5 mm and random positional error of 1.0 mm exhibit minor dose variations, only the dose distributions for the plans with positional errors of ±1.0 and 2.0 mm and random positional error of 2.0 mm are shown.

The reference plan yielded similar dose distributions for the two TPSs, and gEUD sensitivity between the systematic MLC close and open errors was not significantly different except for some results of brain metastasis. The reference treatment plans generated were consistent between the two TPSs within an average D_mean_ of 1.8% to PTV (Planning Target Volume) and within an average OAR of 3.5% to the dose constraints, as shown in Table 2. Figure 3 shows an example of dose distribution and DVH for a treatment plan considering systematic MLC positional errors for spinal metastasis. As in this case, the dose distribution of the clinical treatment plan using systematic close/open and random errors with respect to the MLC position changed as the positional error increased.

### 3.2 Changes in gEUD for MLC positional error

**FIGURE 4:**
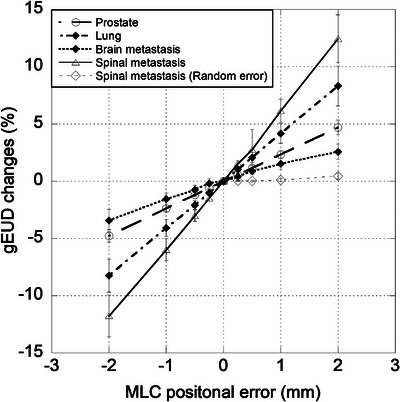
Rate of gEUD change with MLC systematic positional error for prostate cancer (78 Gy/39 fraction), lung cancer (52 Gy/4 fraction), brain metastasis (42 Gy/10 fraction), and spinal metastasis (30 Gy/10 fraction), and with MLC random positional error for spinal metastasis (30 Gy/10 fraction). Each plot shows the average values and standard deviations of 20 cases.

In all cases, a linear relationship was obtained between the rates of change in gEUD for MLC systematic and random positional errors. As shown in Figure 4, the rate of gEUD change was linearly related to the systematic close/open positional error. A linear relationship was obtained for all combinations of TPS, treatment site, and fraction size (*r* = 0.9605−1.000 and 0.9964−1.000 for Monaco and RayStation, respectively). The rate of gEUD change was also linearly related to the random positional error for spinal metastasis (*r* = 0.9749−0.9855 and 0.8979−0.9606 for Monaco and RayStation, respectively). For all cases of spinal metastasis, the gEUD sensitivity for random positional error was 0.12 ± 0.08%/mm and 0.07 ± 0.05%/mm (for Monaco and RayStation, respectively), and 6.67 ± 1.18%/mm, 8.94 ± 3.16%/mm (Monaco and RayStation, respectively) for systematic positional error. It was found that the MLC systematic positional error had a significant effect on dose error. Therefore, the gEUD dose sensitivities described as follows were investigated for systematic positional errors.

**FIGURE 5:**
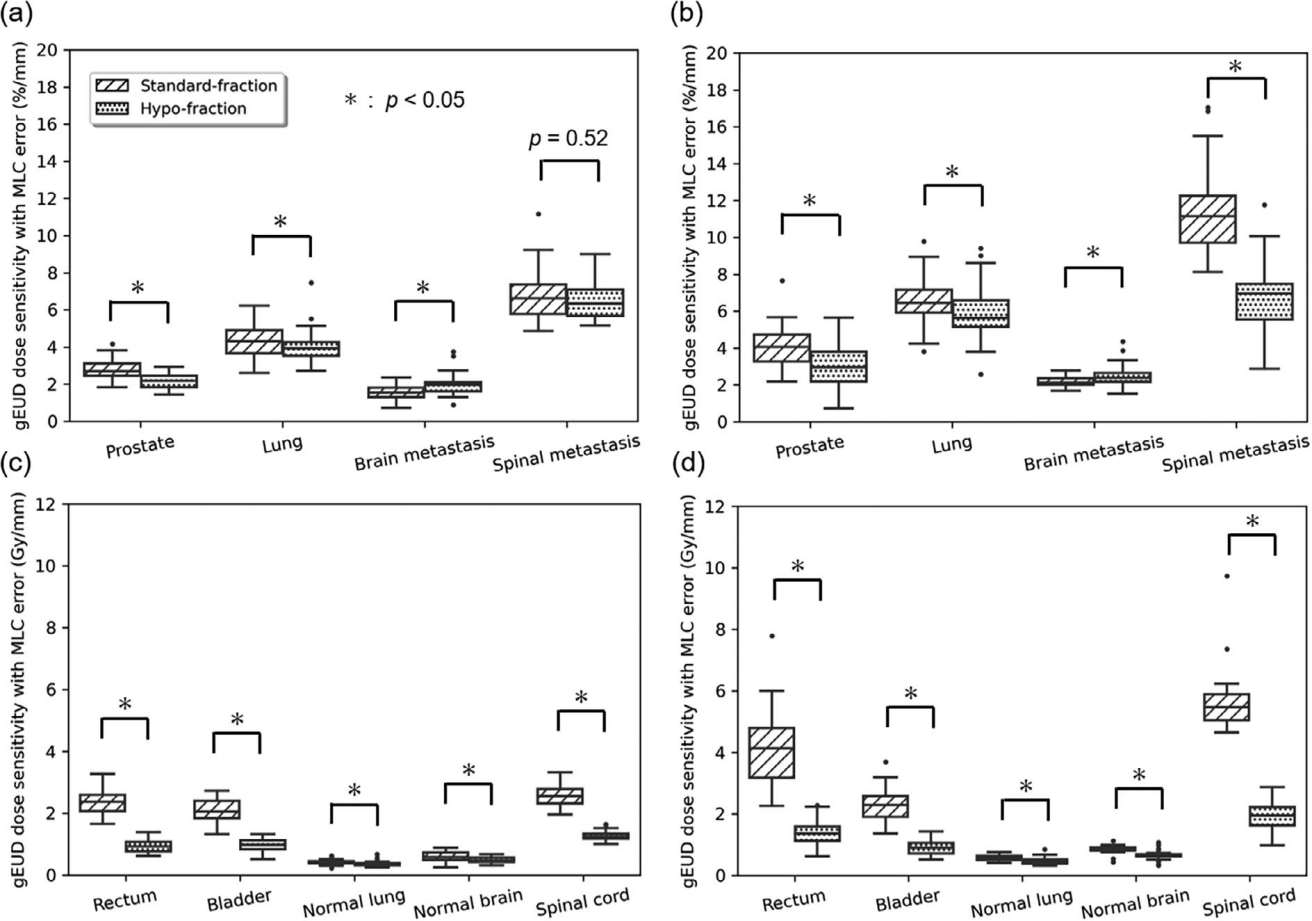
Box plots showing the relationships of generalized equivalent uniform dose (gEUD) sensitivity with MLC systematic positional error for treatment site, TPS, and fraction size. (a) gEUD sensitivity for target volume using Monaco, (b) gEUD sensitivity for target volume using RayStation, (c) gEUD sensitivity for organs at risk (OARs) using Monaco, and (d) gEUD sensitivity for OARs using RayStation. For each box, the centerline indicates the median, the top and bottom of the box indicate the 25th and 75th percentiles, respectively, and the dot plot indicates the outlier. The *p*‐value indicates the significant difference in gEUD sensitivity for standard fractions and hypofractions.

The gEUD sensitivity was most dependent on the treatment site for systematic positional errors, followed by the TPS and fraction size. Table S1 shows the results of the Shapiro–Wilk test used as a normality assessment test of gEUD sensitivity. Subsequent significance tests were examined using nonparametric tests for groups that were not normal based on the normality assessment in Table S1. There was no clear significant difference in gEUD sensitivity for FF and FFF beams. Therefore, the gEUD sensitivities for FF and FFF beams were analyzed together. Figure 5a,b show the gEUD sensitivity plots for the target volumes using Monaco and RayStation, respectively, and Figure 5c,d show the gEUD sensitivity for OARs. For Monaco, the maximum gEUD sensitivity was 6.7 ± 1.2%/mm for spinal metastasis and 1.9 ± 0.7 Gy/mm (target volume and spinal cord, respectively). The smallest were brain metastasis for target volume (1.7 ± 0.5%/mm) and normal lung for OARs (0.4 ± 0.1 Gy/mm). For RayStation, the maximum gEUD sensitivity was 8.9 ± 3.2%/mm for spinal metastasis and 3.8 ± 2.0 Gy/mm (target volume and spinal cord, respectively). The minimum was brain metastasis for target volume (2.3 ± 0.4%/mm) and normal lung for OARs (0.5 ± 0.1 Gy/mm). The highest gEUD sensitivity for spinal metastasis was found in both the target volume and OARs.

In comparison with the TPSs, the overall gEUD sensitivity was significantly higher for RayStation compared with that for Monaco (*p* < 0.05). There was no significant difference in gEUD sensitivities between the two TPSs for the bladder (*p* = 0.26), but the gEUD sensitivity was significantly higher using RayStation than using Monaco at sites other than the bladder for OARs (*p* < 0.05). As shown in Figure 5, the differences in average gEUD dose sensitivity between TPSs were 2.3, 1.8, 1.1, and 0.6%/mm (spinal metastasis, lung cancer, prostate cancer, and brain metastasis, respectively) in target volume, 1.8, 1.1, 0.1, and 0.2 Gy/mm (spinal cord, rectum, bladder, normal lung, and normal brain, respectively) in OARs. The gEUD sensitivity differed significantly between the two TPSs, with the largest differences for brain metastasis relative to the target volume and spinal cord relative to the OARs.

In comparison, for fraction size, the gEUD sensitivity in the standard fraction tended to be larger than that in the hypofraction for treatment sites, excluding brain metastasis (*p* < 0.05). As shown in Figure 5, only in the case of brain metastasis to the target volume, the average gEUD sensitivity was significantly smaller for the standard fraction than for the hypofraction (*p* < 0.05). For spinal metastasis, which yielded the highest gEUD sensitivity in the comparison of treatment sites, the average difference in fraction size relative to target volume was 0.2%/mm for Monaco and 4.8%/mm for RayStation. For the spinal cord, the difference in average gEUD sensitivity between fraction sizes was 1.3 Gy/mm for Monaco and 3.7 Gy/mm for RayStation. In particular, the gEUD sensitivity tended to be higher for standard fraction than for hypo fraction in OAR.

### 3.3 Relationship between complexity metrics and gEUD sensitivity

**FIGURE 6:**
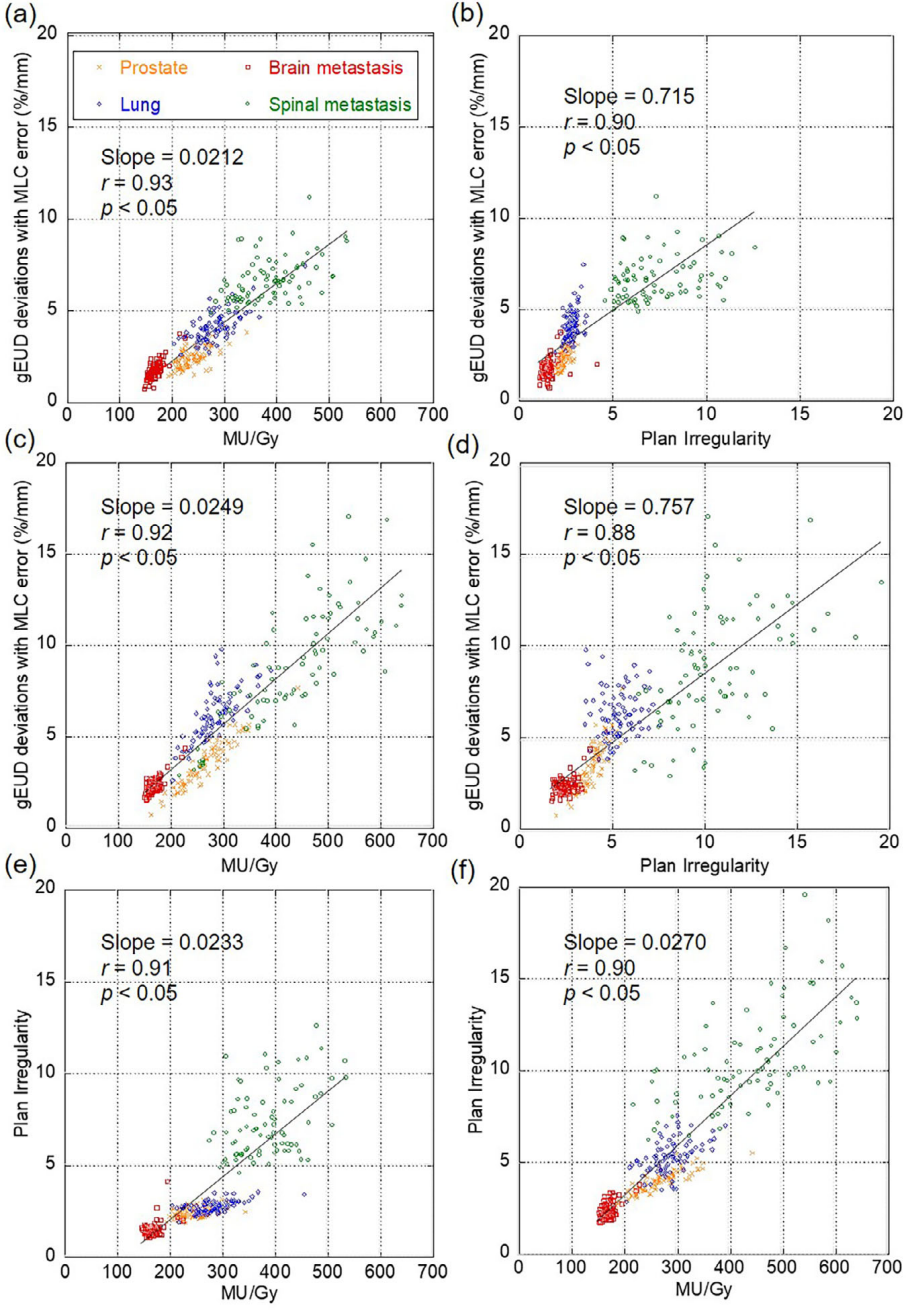
Scatter plots showing the correlation between gEUD sensitivity and complexity metrics. Each plot and correlation show the variations of the (a) gEUD sensitivity to MU/Gy and (b) gEUD sensitivity to plan irregularity (PI) for Monaco, and (c) gEUD sensitivity to MU/Gy and (d) gEUD sensitivity to PI for RayStation, (e) PI to MU/Gy for Monaco and (f) PI to MU/Gy for RayStation. The plots show the average gEUD sensitivity to the complexity metrics for reference plans due to MLC systematic positional errors between metrics. The symbol *r* is Spearman's correlation coefficient, and the *p*‐value indicates a significant difference.

For any treatment site, gEUD sensitivity yielded strong correlations with the complexity metrics. Figure 6 shows the correlation between gEUD sensitivity and complexity metrics. As shown in Figure 6, MU/Gy and gEUD sensitivity yielded a strong correlation coefficient for both TPSs (*r* = 0.93 and 0.92, Monaco and RayStation, respectively). gEUD sensitivity also yielded a strong correlation for PI (*r* = 0.90 and 0.88, Monaco and RayStation, respectively). As shown in Figure 6e,f MU/Gy and PI yielded a strong correlation with both TPSs (*r* = 0.91 and 0.90, for Monaco and RayStation, respectively). These results suggested that the complexity metrics might be a surrogate of the gEUD sensitivity.

**FIGURE 7:**
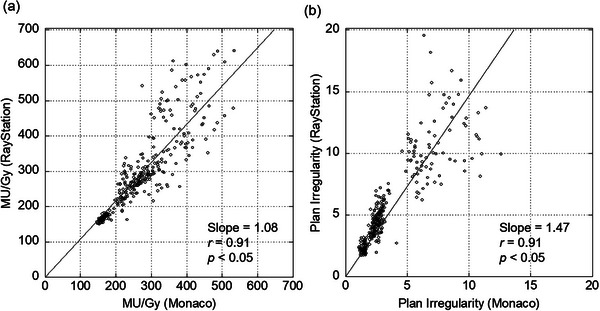
Scatter plots and linear regression lines showing correlations between the complexity metrics of the two TPSs. Each plot and correlation show (a) MU/Gy and (b) PI between TPSs. The symbol *r* is the Spearman's correlation coefficient and the *p*‐values indicate significant differences.

Complexity metrics between TPSs were comparable for MU/Gy but different for PI. As shown in Figure 7, the comparison of complexity metrics between TPSs showed high linearity (*r* = 0.91) between both complexity metrics. As shown in Figure 6, a plot of gEUD sensitivity versus MU/Gy showed slopes of 0.0212 and 0.0249 (Monaco and RayStation, respectively). In the plot of gEUD sensitivity versus PI, the slopes were 0.715 and 0.757 (Monaco and RayStation, respectively). The slopes were comparable between the TPSs, suggesting that MU/Gy and PI provide comparable gEUD sensitivity independently of TPS. The slopes of the scatter plots between the complexity metrics for the different TPSs shown in Figure 7 were 1.08 and 1.47 (MU/Gy and PI, respectively). MU/Gy correlated with gEUD sensitivity independently of TPS, but the slope of PI was slightly higher for RayStation. Despite differences in complexity metrics between TPSs, MU/Gy was suggested to be more strongly correlated with gEUD sensitivity than PI, independent of TPS.

### 3.4 Determination of tolerances required for MLC positional accuracy control

The minimum MLC positional accuracies required by the MLC positional accuracy control were found to be 0.63, 0.34, 1.02, and 0.28 mm for prostate cancer, lung cancer, brain, and spinal metastases, respectively. Table 3 lists the MLC positional accuracy values that can maintain ±2% of the rate of gEUD change relative to the target volume for the combination of the treatment site and fraction size. The combination with the smallest tolerance required by the MLC positional accuracy control was spinal metastasis for the standard fraction using RayStation TPS. In particular, the results showed that the tolerance of the MLC positional accuracy varied depending on the treatment site.

**TABLE 3:** Comparison of the tolerance of the positional accuracy of multi‐leaf collimators (MLCs) in clinical situation combinations.

	Positional accuracy of MLC required for accuracy control (mm)			
	Monaco		RayStation	
Treatment site	Standard fraction	Hypofraction	Standard fraction	Hypofraction
Prostate	0.75	0.95	0.63	0.78
Lung cancer	0.46	0.48	0.34	0.36
Brain metastasis	1.28	1.02	1.25	1.17
Spinal metastasis	0.32	0.31	0.28	0.42

## 4 DISCUSSIONS

To our knowledge, this is the first study to examine dose errors due to MLC positional errors related to treatment planning complexity in VMAT for multiple treatment site and fraction size combinations using different TPSs (Monaco, RayStation). This study showed that the dose error due to MLC positional error varied significantly according to the treatment site and that the dose error also varied based on the combination of TPS and fraction size. Furthermore, the dose error due to MLC position error was suggested to vary depending on the complexity metrics. In particular, the tolerances of MLC positional error vary considerably depending on the treatment site. Therefore, we proposed the use of a complexity metric, such as MU/Gy, as a predictive indicator for MLC positional accuracy to control MLC positional accuracy for each treatment site. This study is expected to reduce dose errors in patients in the treatment planning stage and to enable easier and more detailed MLC positional accuracy management than by managing MLC quality from the complexity metrics.

The dose error due to MLC positional error varies with treatment site and TPS, as in previous studies, and a new finding is that it varies with fraction size. Table 4 summarizes the reports of the previous studies regarding the dose sensitivity to MLC positional error obtained in limited clinical situations. The results of the current study showed that the dose errors for prostate cancer were 2.5 and 3.5%/mm (Monaco and RayStation, respectively). For RayStation, the dose error for prostate cancer was slightly higher; however, for Monaco, it was consistent with the previous studies.^4^, ^34^ Saito et al.^35^ assessed MLC error sensitivity using dosimetry devices for head and neck, lung, and prostate cancers, and found that dose errors varied as a function of the treatment site. The results of this study also showed that the dose error due to the MLC positional error varied depending on the treatment site. Tatsumi et al.^36^ examined the difference in gamma pass rates owing to differences in TPS using dosimetry devices. The gamma pass rates of the measured dose distributions calculated using the criteria of 2% dose difference were 68.9, 89.5, and 99.3% (Monaco, SmartArc, and Ergo, respectively) at 1 mm leaf offset. These results are consistent with those of this study in that the dose errors due to MLC positional errors differ as a function of the TPS. The difference in dose errors due to the MLC positional errors may be attributed to the different leaf sequences in each TPS, even if the linear accelerator is the same. Monaco applied jaw tracking, but RayStation did not; thus, the MLC sequence was different. Additionally, RayStation did not have the sliding window function, which may also manifest itself as a difference in the MLC sequence. We also found that the dose error changed depending on the fraction size, as shown in Figure 5. This may be related to the fact that the MLC sequence became more complex for standard fraction than for hypofraction, depending on the fraction size. Therefore, it is possible that different MLC sequences depending on the treatment site and TPS or fraction size may result in different susceptibilities to dose errors.

**TABLE 4:** Summary of dosimetric impacts due to MLC positional errors assessed in previous studies.

Study	Treatment site	TPS	Beam modality	Fraction size	Key findings
Mu et al.^6^ (2008)	Head and neck (H&N) (17 patients)	Pinnacle	FF	2.0 Gy	Dose sensitivities for systematic error were 4%/mm (simple plan) and 8%/mm (complex plan)
Rangel and Dunscombe^7^ (2009)	H&N, prostate (14 patients)	Eclipse	FF	2.0 Gy	Dose sensitivities for systematic error were 5.7%/mm (H&N) and 2.7%/mm (prostate).
Oliver et al.^4^ (2010)	H&N (8 patients)	Eclipse	FF	2.0 Gy	Dose sensitivities for systematic error were 3.2%/mm (RapidArc), 2.9%/mm (simple intensity‐modulated radiotherapy (IMRT) plan) and 9.6%/mm (complex IMRT plan).
Oliver et al.^1^ (2011)	Prostate (10 patients)	Eclipse	FF	2.0 Gy	Dose sensitivities for systematic error were 7.2%/mm and 8.2%/mm (close and open, respectively).
Bai et al.^2^ (2013)	Nasopharyngeal carcinoma (6 patients)	Pinnacle	FF	1.8−2.1 Gy	Dose sensitivities for systematic error were 3.4%/mm (PTV) and 8.7%/mm (parotid glands).
Katsuta et al.^34^ (2016)	H&N, prostate	Monaco	FF	2.0 Gy	Dose sensitivity for systematic error were 2.6%/mm (H&N) and 2.4%/mm (prostate) for systematic MLC positional error.
Blake et al.^3^ (2017)	Lung SBRT (18 patients)	Pinnacle	Unknown	12 Gy, 18 Gy, 20 Gy	Dose sensitivities for systematic error were −3.7% and 0.8% (close and open, respectively) for ssIMRT and −4.0% and 1.1% (close and open, respectively) for VMAT at 1 mm systematic MLC positional error.
Feng et al.^9^ (2020)	NSCLC (10 patients)	Pinnacle	FF	10.0 Gy	Dose deviations were −0.5, −3.4, −3.6, −4.8, and 2.6 Gy/mm for random, left shift, right shift, systematic close, and systematic open error, respectively. Dose sensitivity: auto planning < manual planning.

Abbreviations: FF, flattening fliter; NSCLC, non‐small cell lung cancer; SBRT, stereotactic body radiation therapy; TPS, treatment planning system.

The variations in gEUD sensitivity across clinical situations can be explained by the complexity metrics. The gEUD sensitivity and the complexity metrics were strongly correlated (*r* = 0.88‐0.93). Plans with increased complexities have larger gEUD sensitivities. The stronger correlations between the gEUD sensitivity and complexity metrics were observed even though the plan optimizer and MLC sequencer (i.e., different TPSs) were different. This suggests that the complexity metrics could be a predictive indicator of the dose error caused by the MLC positional error in any clinical situation. It may also be related to the complexity of the MLC sequence with respect to the gEUD sensitivity outliers shown in Figure 5. Moreover, a linear correlation was also observed between the two complexity metrics (*r* = 0.90, 0.91). This is probably because the MLC aperture shapes are smaller or more irregular in higher MU/Gy plans. In comparison, MU/Gy yielded slightly higher correlation coefficients, thus suggesting that the MU/Gy would be a more important measure. The MU/Gy values were almost equal between the two TPSs in the same patient because the slope of the linear regression line was approximately 1.0 (see Figure 7). Therefore, the same tolerance value of MU/Gy can be used in the two TPSs. Differences in PI values between the two TPSs in the same patient were also observed. This result indicates that MLC aperture shape was more irregular in the RayStation plans. This would be one possible reason for the slightly different gEUD sensitivity between the two TPSs.

Tolerances for MLC positional accuracy vary by treatment site, and appropriate tolerances should be established for each treatment site with reference to the treatment planning complexity metric. You et al.^8^ reported that for spinal metastasis, the acceptable range for systematic MLC positional error was ≤0.3 mm. Several authors have reported an acceptable range of MLC positional error of 0.3–1.0 mm^2^, ^4^, ^12^, ^36^ and AAPM TG142^37^ recommends an MLC positional error of 1 mm or less. The acceptable range of positional accuracy of the MLC varied, especially depending on the treatment site. Differences in tolerance of MLC positional accuracy due to differences in TPS and fraction size were not clinically problematic. Therefore, depending on the treatment site, each facility should establish an acceptable range of MLC positional accuracy. The MLC positional accuracy management is possible by performing the DMLC relative output test proposed by Lasasso et al.^38^ Furthermore, an acceptable range of the complexity metric (i.e., MU/Gy), can be determined from the linear relationship between the complexity metrics and gEUD sensitivity. The MU/Gy is easy to control during treatment planning and simple to analyze, and the dose delivery uncertainty in MLC positional accuracy can be easily controlled at any facility.

This study was associated with several limitations. First, it focused only on the MLC positional error. In practice, dose errors are likely to occur because of the mechanical accuracy limits of radiotherapy devices caused by the positional accuracy of the output MU and gantry. Another limitation is that this study was based on simulations rather than detector dosimetry. In addition, as the treatment plan was generated by a single planner, the possibility of bias in the results cannot be disregarded. In the future, a combined study in conjunction with dosimetry, including error factors other than the MLC positional errors, will make it possible to evaluate the dose error under more clinically relevant conditions. Furthermore, it is also necessary to verify the impact of dose verification on the results by applying and controlling MLC tolerances using the complexity metrics for each treatment site.

## 5 CONCLUSIONS

The dose sensitivity caused by the MLC positional error varied depending on the treatment site, TPS, and fraction size. The dose error owing to the MLC positional error depended on the MLC sequences, which varied with the complexity of the plan. The minimum allowable positional errors for MLC were 0.63, 0.34, 1.02, and 0.28 mm (for prostate, lung, brain, and spinal metastasis, respectively). MLC positional accuracy depended on a combination of several clinical situations that varied by the complexity metric. By applying and controlling the complexity metric such as MU/Gy at each facility, it may be possible to determine the optimal MLC tolerance and reduce dose errors due to MLC positional errors.

## AUTHOR CONTRIBUTIONS

Hiromi Enomoto and Yukio Fujita conceived the idea for the study. Saki Matsumoto made major contributions to data analysis and interpretation. Yujiro Nakajima, Miyuki Nagai, Ayako Tonari, and Takeshi Ebara contributed to the interpretation of the results. Hiromi Enomoto prepared the manuscript and Yukio Fujita oversaw the conduct of this study. All authors approved the submitted manuscript and agreed to accept responsibility for any part of this study.

## CONFLICT OF INTEREST STATEMENT

The authors declare that they have no known competing financial interests or personal relationships that could have appeared to influence the work reported in this paper.

## Supporting information

Supporting Information

## Data Availability

The data that supports the findings of this study are available in the supplementary material of this article.
