# Supplementary material for: Dosimetric impact of MLC positional errors on dose distribution in IMRT
Source: J Appl Clin Med Phys. 2023 Sep 18;25(2):e14158. doi: 10.1002/acm2.14158 (PMC10860456; doi:10.1002/acm2.14158)
Supplement: Supplementary file 1 — Supporting Information [file ACM2-25-e14158-s001.docx]

**Supplemental data**

**Table S1** Normality evaluation by *p*-value obtained with the Shapiro–Wilk test for generalized equivalent uniform dose (gEUD) sensitivity.

|  |  | Monaco | | RayStation | |
| --- | --- | --- | --- | --- | --- |
|  |  | Standard  fraction | Hypofraction | Standard  fraction | Hypofraction |
| Prostate | PTV | 0.46 | 0.54 | 0.16 | 0.69 |
|  |  | 0.11 | | 0.35 | |
|  | Rectum | 0.49 | 0.46 | 0.06 | < 0.05 |
|  | Bladder | 0.44 | 0.51 | 0.74 | 0.53 |
| Lung | PTV | 0.93 | < 0.05 | 0.93 | 0.30 |
|  |  | < 0.05 | | 0.85 | |
|  | Normal lung | 0.11 | < 0.05 | 0.35 | < 0.05 |
| Brain metastasis | PTV | 0.76 | < 0.05 | 0.25 | < 0.05 |
|  |  | < 0.05 | | < 0.05 | |
|  | Normal brain | 0.08 | 0.71 | < 0.05 | < 0.05 |
| Spinal metastasis | PTV | < 0.05 | < 0.05 | < 0.05 | 0.08 |
|  |  | < 0.05 | | 0.38 | |
|  | Spinal cord | 0.61 | 0.09 | < 0.05 | 0.99 |

Table S1 shows the results of the Shapiro–Wilk test used as a normality assessment test of gEUD sensitivity. Subsequent significance tests were examined using nonparametric tests for groups that were not normal based on the normality assessment in Table S1.
